# Adhesion obstacles effect on PIPAC patients with primary unresectable or recurrent platinum-resistant peritoneal metastasis from ovarian cancer

**DOI:** 10.1515/pp-2024-0011

**Published:** 2025-11-10

**Authors:** Christos Iavazzo, Ioannis D. Gkegkes

**Affiliations:** Gynaecological Oncology Department, Metaxa Cancer Hospital, Piraeus, Greece; Athens Colorectal Laboratory, Athens, Greece; Department of Colorectal Surgery, 9553 Royal Devon and Exeter NHS Foundation Trust, Exeter, UK

To the Editor,

With great deal of interest we read the article entitled “Outcome of patients with peritoneal metastasis from ovarian cancer treated with pressurized intraperitoneal aerosol chemotherapy (PIPAC)” by Foslund et al. [1].

The authors presented a retrospective analysis of a cohort of 23 patients with primary unresectable or recurrent platinum-resistant peritoneal metastasis from ovarian cancer which showed a median overall survival of 8.2 months, a major/complete histological response of 32 %, and three patients with severe adverse reactions. A recent systematic review that PIPAC is safe and effective for palliative purposes, with a good pathological tumour response and quality of life as well as low morbidity and mortality rate [2]. However, many platinum-resistant tumors have proven unresponsive, and multimodal therapy is beneficial in such cases including surgery, chemotherapy, and targeted therapy [3]. Could Foslund et al. inform us how palliative chemotherapy and targeted therapy affected their results regarding the survival and histological response?

One of the main disadvantages of PIPAC is that adhesions secondary to surgery create obstacles to aerosol diffusion. We would also like to ask Foslund et al. whether they have data in their cohort regarding the patients who had or did not have adhesion obstacles effect. Was there any further difference affecting survival or histological response?

Once again, we would like to thank the authors for their contribution.
